# Synthesis, Cytotoxicity, DNA Binding and Apoptosis of Rhein-Phosphonate Derivatives as Antitumor Agents

**DOI:** 10.3390/ijms14059424

**Published:** 2013-04-29

**Authors:** Man-Yi Ye, Gui-Yang Yao, Jing-Chen Wei, Ying-Ming Pan, Zhi-Xin Liao, Heng-Shan Wang

**Affiliations:** 1 State Key Laboratory Cultivation Base for the Chemistry and Molecular Engineering of Medicinal Resources, School of Chemistry & Chemical Engineering of Guangxi Normal University, Guilin 541004, China; E-Mails: yemanyi880706@163.com (M.-Y.Y.); yaoguiyangmayu@126.com (G.-Y.Y.); 2 College of Pharmacy, Guilin Medical University, Guilin 541004, China; E-Mail: jingchenwei@163.com; 3 Department of Pharmaceutical Engineering, School of Chemistry and Chemical Engineering, Southeast University, Nanjing 211189, China; E-Mail: zxliao@seu.edu.cn

**Keywords:** rhein, phosphonate, synthesis, cytotoxicity, apoptosis, DNA binding

## Abstract

Several rhein-phosphonate derivatives (**5a**–**c**) were synthesized and evaluated for *in vitro* cytotoxicity against HepG-2, CNE, Spca-2, Hela and Hct-116 cell lines. Some compounds showed relatively high cytotoxicity. Especially compounds **5b** exhibited the strongest cytotoxicity against HepG-2 and Spca-2 cells (IC_50_ was 8.82 and 9.01 μM), respectively. All the synthesized compounds exhibited low cytotoxicity against HUVEC cells. Further experiments proved that **5b** could disturb the cell cycle in HepG-2 cells and induce apoptosis. In addition, the binding properties of a model conjugate **5b** to DNA were investigated by methods (UV-Vis, fluorescence, CD spectroscopy). Results indicated that **5b** showed moderate ability to interact ct-DNA.

## 1. Introduction

α-Aminophosphonic acids (APAs) and their derivatives are an important class of compounds that exhibited intriguing biological activities [1–10]. A number of potent antibiotics, herbicides, antitumor agents [11–15] and enzyme inhibitors [3] are APAs or their derivatives. Moreover, these compounds have been evaluated as inhibitors of matrix metalloproteinases MMP-1, MMP-2, MMP-3 and MMP-8 [16–18]. Some derivatives were found to trigger apoptotic cell death in DOHH-2 cells [19–21]. Especially their negligible mammalian toxicity and the fact that they very efficiently mimic aminocarboxylic acids makes them extremely important antimetabolites, which compete with their carboxylic counterparts for the active sites of enzymes and other cell receptors [4,22–24]. In recent years, there has been growing interest in the antitumor activities studies on the APAs conjugates [25–27], owing to the important role of APAs in living systems as analogs of natural α-amino acids. However, there are far fewer reports about reducing toxicity of derivatives by APAs.

Rhein, a main constituent of rhubarb, is a well-characterized anti-inflammatory agent which has been used for treatment of inflammatory diseases such as osteoarthritis, diabetic nephropathy, *etc*. [28–30]. It has also been reported to display inhibitory effects on the proliferation of human breast, colon, lung, NPC [31], CNS, and glioma cancer cells [32–34]. Especially some rhein derivatives induce apoptosis in several cancer cell lines such as human colonic adenocarcinoma cells, promyelocytic leukemia cell (HL-60), human nasopharyngeal carcinoma cells, human breast cancer cells and cervical cancer Ca Ski cells [35,36]. Anthraquinone-based drugs have been shown to possess strong antiperliferative properties, such as mitoxantrone, ametantrone, and anthracycline antibiotics (daunorubicin and doxorubicin), and are used for treatment of varied oncology [37]. However, these clinically used drugs also suffered from frequent cardiotoxicity that limited their applicability [38]. Recently, some analogs of APAs had been synthesized to investigate their antioxidant activities [10]. It was found that antioxidant molecules and enzymes can potentially limit the oxidative injury, but most of them are not readily internalized within myocardial cells, or they cannot reach the right cell compartment to exert their protective effect [39,40]. In addition, Łukasz Berlicki suggested that the N–C–P molecular fragment play an important role in the enhancement of antitumor or antivirus activity of leading compounds and its chemistry offer many possibilities for their structural modification [41].

Based on the above considerations, we postulated that the novel conjugates enhanced the cytotoxicity and reduced the induced toxic on human normal cells. In this paper, we report (a) the synthesis of the rhein derivatives with APAs; (b) DNA binding of **5b**; (c) *in vitro* anti-cancer activity and cell selectivity of synthesized conjugates; (d) and the mechanism of how the novel conjugates killed HepG-2 cells.

## 2. Results and Discussion

### 2.1. Chemistry

**Scheme 1:**
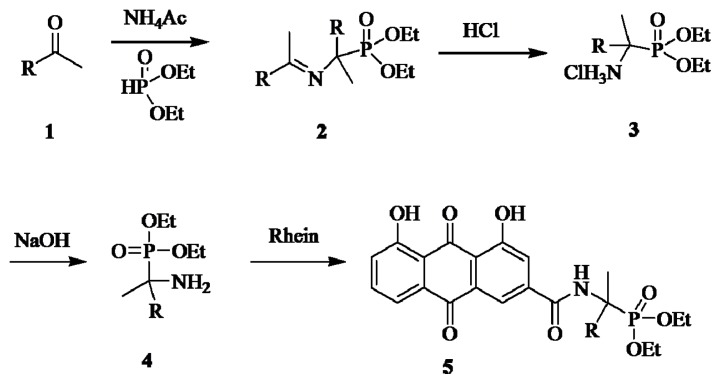
General synthetic route for compound **5a**–**c**.

The syntheses of the phosphonate conjugates **5a**–**c** were achieved by a convenient procedure shown in Scheme 1.

*O*,*O*′-dialky {[N-(phenylmethylene)-α-amino]-α-(substituted phenyl)methyl}phosphonates **2**, obtained by reacting of substituted aromatic ketones **1** with ammonium acetate and *O*,*O*′-dialkyl phosphite, were converted easily to *O*,*O*′-dialkyl α-amino-α-(substituted phenyl)methyl phosphonate **4** via hydrolysis [42,43]. The α-methyl-substituted aminophosphonates **4** were then coupled with rhein to provide phosphonate conjugates **5** in satisfactory yields.

All of compounds in the series **5** were obtained as yellowish solids after column chromatography. Their structures were fully characterized by ^1^H-NMR. For example, the corresponding ^1^H-NMR spectrum showed the carbonyl group leads to a downfield shift of the two phenolic hydroxyls. The two methyleneoxy (OCH_2_CH_3_) groups attached with phosphorus appear as three multiplets at 3.30–4.30. The chemical shifts of the two methyl (OCH_2_CH_3_) hydrogens were different due to the low rate of environmental exchange caused by the slow rotation of the P–C bond. The structures of **5a** and the other analogs were further confirmed by ^31^P-NMR, EI-MS and elemental analysis.

### 2.2. Cytotoxicity Effects

The cytotoxicity results of all of the compounds against HepG-2, CNE, Spca-2, Hela, Hct-116 tumor cell lines and HUVEC normal cells lines are listed in Table 1. From these data, all conjugates exhibited good to moderate cytotoxicity. Especially, compound **5b** exhibited the strongest cytotoxicity against HepG-2 cells with IC_50_ 8.82 ± 0.95 μM, which is even higher than that of fluorouracil. It was because of more hydrophobicity of 5b compared to 5a and 5c. The partition coefficient values were calculated by using the HyperChem Professional program, and the values (logP) of **5a**, **5b** and **5c** were 2.37, 3.05, 2.75, respectively. However, all compounds **5a**–**c** show low cytotoxic effect on the Hela cells. These results showed that these conjugates have selective and significant effect on the cell lines. For comparison, activity of compounds against normal cells (HUVEC) was also examined (Table 1). The results indicated that the anti-proliferative activity of some compounds against cancer cells was much higher than normal cells and maybe one cargo superior to anthracene.

### 2.3. DNA Binding

Although some evidences suggest that other biological targets, including RNA or proteins, may play some important roles in the daunorubicin and mitoxantrone binding, it is generally accepted that DNA is the primary target [45,46]. Similarly, the interactions between small molecules and DNA are believed to be one of the primary action mechanisms of the antitumor activity. The DNA replication in tumor cells can be blocked via the intercalations of the small molecule between the base pairs of DNA [47,48]. Generally, the active compounds show an approximately planar structure and some hydrophobic characteristics to maximize the intercalations. To investigate their binding properties to DNA, several analytical methods, including UV-Vis, fluorescence and circular dichroism spectroscopies were performed.

#### 2.3.1. UV-Vis Absorption Spectral Analysis

**Figure 1:**
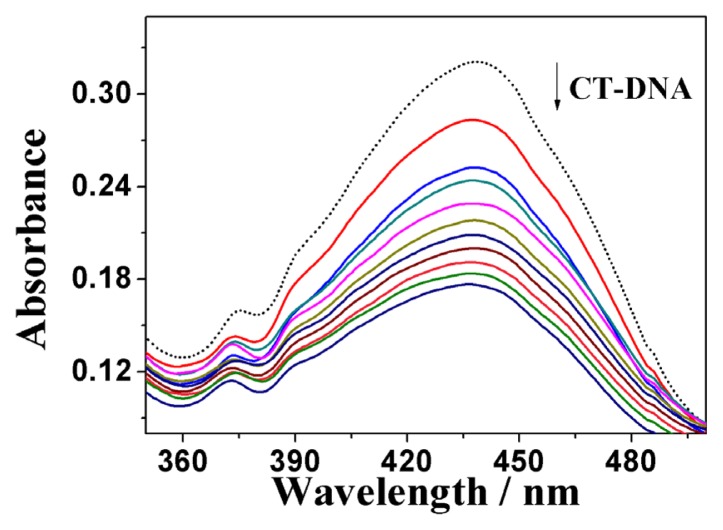
UV-Vis absorption spectra of complex **5b** in the absence (---) and presence (—) of ct-DNA with increasing [DNA]/[**5b**] ratios in the range from 1:1 to 10:1.

The UV-Vis absorption spectroscopy was primarily employed to probe the binding modes of **5b** to the calf thymus DNA (ct-DNA). Rhein belongs to the same class of coplanar anthraquinones as daunorubicin and mitoxantrone, which have been in clinical use over 30 years for the treatment of various tumors [49], which is one kind of DNA targeting agent. Their function as noncovalent DNA binders is generally believed to be essential for their activity. The inherent absorbance of **5b** allowed us to investigate its interaction with ct DNA by absorption spectroscopy. The UV-vis absorption spectra of **5b** in the absence and presence of ct-DNA are shown in Figure 1 (for **5a** and **5c**, see Figures S1 and S2). As shown in the figures, the absorbance of **5a**, **5b** and **5c** changed upon addition of DNA. This indicated that DNA is one potential target of **5** as expected. The DNA binding constant K_b_ was calculated by non-linear fitting according to the Equations (1) [50,51], by which the values of K_b_ for **5b** was found to be 1.2 × 10^4^ M^−^, which is higher than that of **5a** (1.05 × 10^4^ M^−^) and **5c** (0.85 × 10^4^ M^−^).

#### 2.3.2. Fluorescence Emission Titration

The binding ability of these complexes to ct-DNA was primarily investigated by competitive binding in which they served as an intercalative binding probe in competition with GelRed. GelRed, which is environmentally safe and ultra-sensitive for DNA staining, is a newly developed DNA intercalator to replace the classic DNA intercalator EB. Furthermore, both GelRed and EB bound with ct-DNA emit characteristic fluorescence at 590 nm upon 350 nm UV light excitation [52].

**Figure 2:**
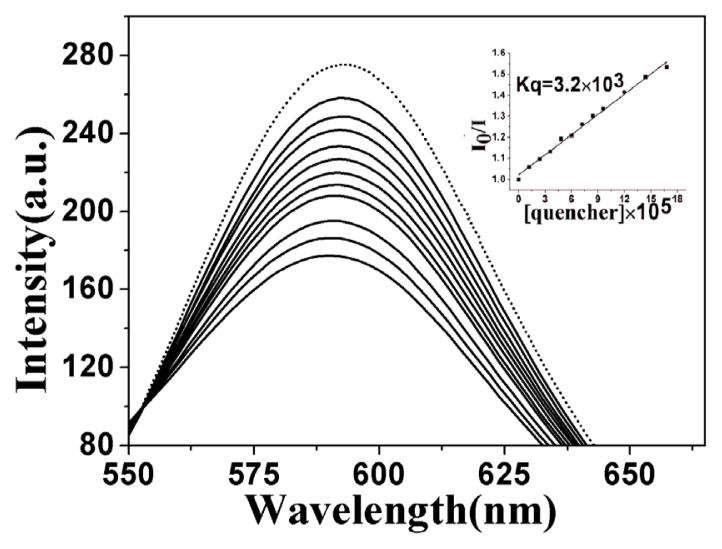
Fluorescence emission spectra of GelRed bound with ct-DNA ([DNA] = 2.0 × 10^−3^ M, [GelRed] = 2.0 × 10^−3^ M) in the absence (dash line) and presence (solid lines) of **5b** with [**5b**]/[GelRed] ratios range from 1:2 to 7.5:1. Inset: linear fitting for quenching constant K_q_ based on Stern-Volmer equation.

In competitive binding experiments, GelRed and ct-DNA solutions were pre-incubated for 30 min to ensure sufficient interactions. The concentration ratio of GelRed to DNA was set at [GelRed]/[DNA] = 1:10 to ensure sufficient binding sites of DNA for GelRed. The emission spectra of the GelRed–ctDNA system in the absence and presence of **5b** were shown in Figure 2 (for that of **5a** and **5c**, see Figures S3 and S4). GelRed–DNA binary solution system gave a characteristic fluorescence emission at around 590 nm when excited at 350 nm, indicating that GelRed molecules intercalated between the adjacent base pairs of DNA and sufficiently prevented fluorescence quenching by polar solvent molecules. The presence of **5b** considerably quenched the fluorescence emission of GelRed with the saturation state achieved at the [**5b**]/[GelRed] ratio of 8:1.

The quenching ability of **5b** to GelRed fluorescence can be quantitively estimated by their respective quenching constant, K_q_, which were derived from the Stern-Volmer quenching equation. The K_q_ value for **5b** was 3.2 × 10^3^, exhibiting similar intensive intercalation to DNA as rhein (3.94 × 10^3^) (Figure S5), stronger intensity than **5a** (2.02 × 10^3^) and **5c** (0.753 × 10^3^) (Figures S3 and S4). Meanwhile, it can also be confirmed that the intercalative binding mode of **5b** to DNA was similar to GelRed [53].

#### 2.3.3. Circular Dichroism Spectra

**Figure 3:**
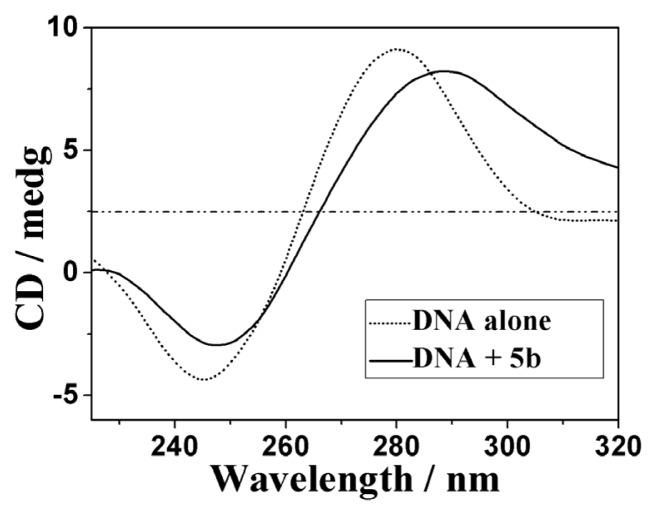
CD spectra of ct-DNA (2 mL solution, 1.5 × 10^−4^ M) in the absence and presence of **5b** (1.5 × 10^−5^ M).

The circular dichroism (CD) is a useful technique to assess whether the nucleic acids undergo conformational changes as a result of complex formation or changes in environmental conditions [54,55]. As indicated in Figure 3, the CD spectra of ct-DNA (1.5 × 10^−4^ M) showed a positive absorption peak at 280 nm and a negative absorption peak at 245 nm due to π–π base stacking and right-hand helicity, respectively. This was consistent with the characteristic B conformation of DNA [56].

As shown in Figure 3, upon the addition of **5b** at a [**5b**]/[DNA] ratio of 1:10, the CD absorption of ct-DNA showed an obvious decrease in the intensities of both the negative and positive absorption bands (for that of **5a** and **5c**, see Figures S6 and S7). The percentage decreases in the maximal DNA positive and negative absorption by **5b** were 9.78% and 32.2%, respectively. It suggested that **5b** might intercalate between the neighboring base pairs of ct-DNA mainly due to the aromatic planarity of the anthraquinones. The decrease in the intensities of both positive and negative bands can usually be observed in the intercalative binding of small molecules to DNA [57]. All these results show clearly that these compounds possess higher DNA binding affinities.

### 2.4. Apoptosis and Cell Cycle Analysis

#### 2.4.1. Apoptosis

**Figure 4:**
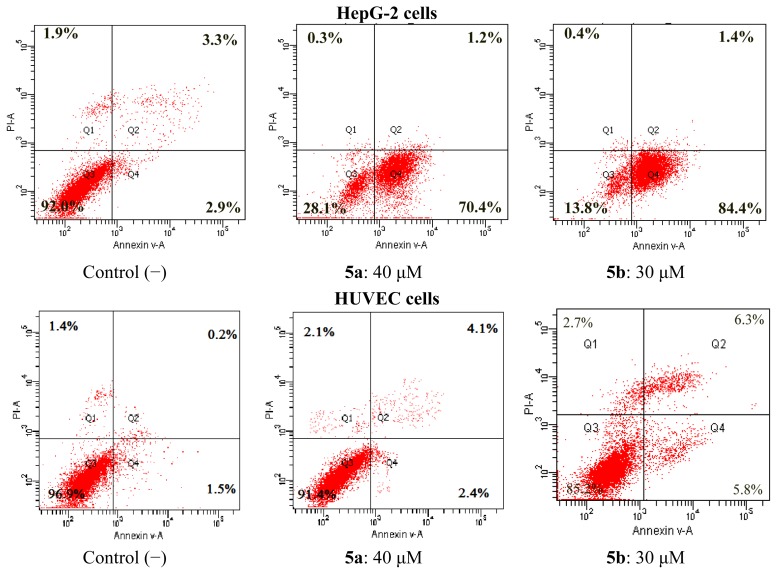
Effect of cyclotriphosphazene compounds on apoptosis of HepG-2 and HUVEC cells. Apoptotic cells were analyzed by flow cytometry, after being stained with annexin V-FITC together with PI. The percentage of cells positive for PI and/or annexin V-FITC are reported inside the quadrants.

Many anthraquinones such as rhein and emodim induce apoptosis [31,33]. Flow cytometry was used to investigate analog-induced apoptosis and cell death. The compounds (**5a**–**c**) with cytotoxic effects were investigated for the effects on the apoptosis of HepG-2 cells. To clarify the mechanism of rhein derivative-induced cell death, we determined both early and late apoptosis using annexin V-FITC and PI (Propidium iodide) labeling of live cells. Annexin V binds to phosphatidylserine, which is exposed on the cell membrane and is one of the earliest indicators of cellular apoptosis. PI is used as a DNA stain for both flow cytometry to evaluate cell viability or DNA content in cell cycle analysis and microscopy to visualize the nucleus and other DNA containing organelles. It can be used to differentiate necrotic, apoptotic and normal cells. In the present study, the apoptotic cell rates were determined for the HepG-2 cells after stimulation for 24 h by the compounds at different concentrations. The graphical values of the results were given in Figure 4. From the results, it was found that these compounds stimulated apoptosis for HepG-2 cells compared to negative (unstimulated cells by compounds) controls. Compounds **5a** and **5b** induced apoptosis in 84.4% and 70.4% of the HepG-2 cells. Compounds **5a** and **5b** are of special interest as these compounds seem to drive HepG-2 cells directly into early apoptosis. Meanwhile, we also investigated for the effects on the apoptosis of HUVEC cells. The result showed that the compounds we have synthesized had no significant apoptotic effects on HUVEC cells.

#### 2.4.2. Cell Cycle Analysis

**Figure 5:**
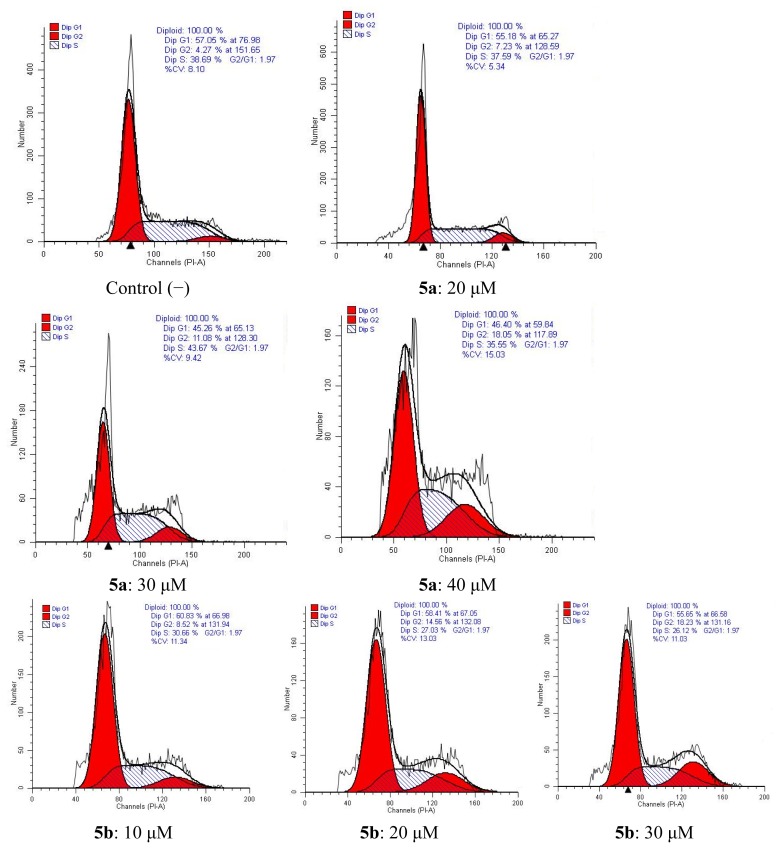
Inhibition of cell cycle progress in HepG-2 cells treated with **5a** and **5b** for 48 h. Cells were fixed with ethanol and stained with PI. Cell cycle distribution was analyzed by flow cytometry.

The cell cycle is the series of events that take place in a cell leading to its division and duplication (replication). The cell cycle consists of four distinct phases: G1 phase, S phase (synthesis), G2 phase (collectively known as interphase) and M phase (mitosis). The G1 stage is the stage when preparation of energy and material for DNA replication occurs. The S stage is the stage when DNA replication occurs. The G2 stage is the stage when preparation for the M stage occurs. The M stage is “mitosis,” and is when nuclear and cytoplasmic division occurs. Flow-cytometric analysis further confirmed tumor cell apoptosis as shown in Figure 5. Cytometric profiles of the PI-stained DNA showed cell cycle arrest of HepG-2 cells treated with **5a** or **5b** for 48 h at different concentrations. G2-phase populations of 7.23%, 11.08%, 18.05% for **5a** at 20 μM, 30 μM, 40 μM were observed compared with the G2-phase population of 4.27% for untreated cells. For compound **5b**, G2-phase populations of 8.52%, 14.56%, 18.23% for **5b** at 10 μM, 20 μM, 30 μM were observed compared with the G2-phase population of 4.27% for control (−) cells.

## 3. Experimental Section

### 3.1. Chemistry

All chemicals (reagent grade) used were commercially available. NMR spectra were measured on a BRUKER AVANCE AV500 spectrometer using TMS as the internal standard. The mass spectra were obtained on a BRUKER ESQUIRE HCT spectrometer. Melting points were determined using an X-4 apparatus and were uncorrected. Elemental analyses were performed on a Vario Micro Cube Elemental instrument and were within 0.4% of the theoretical values.

General Procedure for the Preparation of Rhein α-Aminophosphonic Acids Conjugates **5**

A suspension of aromatic ketones (10 mmol) and ammonium acetate (11 mmol) was stirred for 6 h at reflux. The reaction mixture was filtered to give the white precipitate **2**, to which the diethyl phosphite (5 mmol) was added and the resulting solution was stirred for 6 h at 80 °C. After that, hydrochloric acid (4 mmol) in 30 mL ether was added to the reaction mixture, which was stirred for 2 h at 0 °C to give the precipitate **3**. The precipitate was filtered and washed with ether (3 × 15 mL), then it was added to 10 mL sodium hydroxide solution (10%) and stirred for 30 min at room temperature. Extraction with dichloromethane (3 × 25 mL) and evaporation of the solvent gave the oils of **4**, which was purified by column chromatography on silica gel with ethyl acetate. To a mixture of rhein and HOBt in DMSO (15 mL), the appropriate EDAC was stirred for 15 min at 0 °C. Then **4** was added dropwise with constant stirring at 0 °C, the mixture was stirred at ambient temperature for another 10 h, then washed with brine, dried with anhydrous sodium sulfate and evaporated. The residue was purified by column chromatography on silica gel with petroleum ether/ethyl acetate (3:1, *v:v*) to give the pure title compounds **5** as yellowish solids.

*O,O*′*-Diethyl{[2-(4,5-dihydroxy-9,10-dioxo-9,10-dihydroanthracene)acetamido] (4-tolyl) ethyl} phosphonate* (**5a**). Yield 78%. m.p. 132~134 °C. ^1^H-NMR (500 MHz CDCl_3_) *δ* (ppm):0.88 (t, *J* = 6.5 Hz, 3H, CH_3_), 1.31 (t, *J* = 7.0 Hz, 3H, CH_3_), 2.20 (d, ^3^*J (P*,*H)* = 16.0 Hz, 3H, CH_3_), 2.33 (s, 3H, Ar-CH_3_), 3.72–4.05 (m, 4H, OCH_2_), 7.16–8.16 (m, 10H, Ar-H, 1H, NH), 11.99 (s, 1H, OH), 12.06 (s, 1H, OH). ^31^P{H} NMR(202 MHz, CDCl_3_) *δ* (ppm): 24.18. ESI-MS *m/z*: 560.2 (M+Na)^+^. Anal. Calc. (for C_28_H_28_NO_8_P+H_2_O): C, 62.57; H, 5.25; N, 2.61; Found: C, 60.57; H, 5.44; N, 2.52.

*O,O*′*-Diethyl{[2-(4,5-dihydroxy-9,10-dioxo-9,10-dihydroanthracene)acetamido] (Phenylethyl) ethyl} phosphonate* (**5b**). Yield 71%. m.p. 119~121 °C. ^1^H-NMR (500 MHz CDCl_3_) *δ* (ppm): 0.87 (t, *J* = 6.3 Hz, 3H, CH_3_), 1.39 (t, *J* = 7.1 Hz, 3H, 2×CH_3_), 1.83 (d, ^3^*J (P,H)* = 16.0 Hz, 3H, CH_3_), 2.32–2.81 (m, 4H, OCH_2_), 4.24 (m, 4H, CH_2_), 6.52 (d, *J* = 5.71 Hz, 1H, NH), 7.13–8.04 (m, 10H, Ar-H), 11.95 (s, 1H, OH), 12.01 (s, 1H, OH). ^31^P{H} NMR (202 MHz, CDCl_3_) *δ* (ppm): 26.67. ESI-MS *m/z*: 574.2(M+Na)^+^. Anal. Calc. (for C_29_H_30_NO_8_P+H_2_O): C, 63.15; H, 5.48; N, 2.54; Found: C, 61.16; H, 5.66; N, 2.46.

*O,O*′*-Diethyl {[2-(4,5-dihydroxy-9,10-dioxo-9,10-dihydroanthracene) acetamido] (phenyl) methyl} phosphonate* (**5c**). Yield 78%. m.p. 141~143 °C. ^1^H-NMR (500 MHz CDCl_3_) δ (ppm): 1.23 (t, *J* = 6.9 Hz, 3H, CH_3_), 1.30 (t, *J* = 6.9 Hz, 3H, CH_3_), 2.23 (d, ^3^*J (P*,*H)* = 15.9 Hz, 3H, CH_3_), 3.71–4.06 (m, 4H, OCH_2_), 7.26–8.16 (m, 10H, Ar-H, 1H, NH), 11.98 (s, 1H, OH),12.06 (s, 1H, OH). ^31^P{H} NMR (202 MHz, CDCl_3_) δ (ppm): 24.07. ESI-MS *m/z*: 546.2 (M+Na)^+^. Anal. Calc. (for C_27_H_26_NO_8_P+H_2_O): C, 61.95; H, 5.01; N, 2.68; Found: C, 59.89; H, 5.21; N, 2.59.

### 3.2. Biological Assays

#### 3.2.1. Cytotoxicity of Rhein Derivatives

##### 3.2.1.1. Cell Lines

The following *in vitro* human cancer cell lines were used: HepG 2 (human epidermoid larynx carcinoma), Hela (Henrietta Lacks strain of cancer cells), Hct-116 (human colorectal cells), CNE (human nasopharyngeal carcinoma cells), Spca-2 (human lung adenocarcinoma cell line), HUVEC (human umbilical vein endothelial cells). The cell lines (HepG 2, Hela, Hct-116, CNE, Spca-2, HUVEC) were purchased from the Cell Resource Center of Shanghai Institutes for Biological Sciences, The Academy of Sciences of China.

##### 3.2.1.2. Cell Culture

HepG-2, Hela, Hct-116, CNE, HUVEC cells were cultured in Dulbecco Modified Eagle Medium (DMEM) (Thermo), containing 4.0 mM l-Glutamine and 4,500 mg/L Glucose, supplemented with 10% (*v*/*v*) fetal bovine serum (FBS; HyClone, Logan, UT, USA). Spca-2 cells were cultured in RPMI-1640 medium, containing 2.05 mM l-Glutamine without Calcium nitrate, supplemented with 10% (*v*/*v*) fetal bovine serum (FBS; HyClone). The cell culture media was supplemented with penicillin/streptomycin at 100 Units/mL as adherent monolayers. Cell cultures were kept in a humidified incubator with 5% CO_2_ at 37 °C. Stock solutions were prepared in dimethyl sulfoxide (DMSO) and further dilutions were made with fresh culture medium. The concentration of DMSO in the final culture medium was 1%, which had no effect on the cell viability.

##### 3.2.1.3. MTT Assay

Chemosensitivity was assessed using 3-(4,5-dimethylthiazol-2-yl)-2,5-diphenyl tetrazolium bromide (MTT) assay. Briefly, exponentially growing HepG-2 (2,000–3,000 cells/well), Hela (2,000–3,000 cells/well), Hct-116 (3,000–4,000 cells/well), CNE (2,000–3,000 cells/well), HUVEC (2,000–3,000 cells/well) and Spca-2 (2,000–3,000 cells/well) were seeded into 96-well plates and treated with indicated concentrations of samples for 48h, and then 10 mL of MTT (10 mg/mL) was added. After incubation for 4 h at 37 °C, the purple formazan crystals (*i.e.*, a reduced form of MTT) generated from viable cells were dissolved by adding 100 μL DMSO in each well. The plates were swirled gently for 10 min to dissolve the precipitate, and quantified by measuring the optical density (OD) of the plates at a wavelength of 490 nm on plate reader (TECAN infinite M1000). Each concentration was repeated in three wells and the same experimental conditions were provided for all compounds and MTT analysis was repeated three times for each cell line.

#### 3.2.2. Determination of Apoptosis and Cell Cycle Analysis

##### 3.2.2.1. Apoptosis Analysis

Apoptosis was discriminated with the annexin V-FITC/propidium iodide test. Cells were seeded at 2 × 10^6^/well in 10% FBS-DMEM into 6-well plates, and treated with compounds for 24 h. The cells were washed twice with cold phosphate buffered saline (PBS) and then resuspended in 1× Binding Buffer (0.1 M Hepes/NaOH (pH 7.4), 1.4 M NaCl, 25 mM CaCl_2_) at a concentration of 1 × 10^6^ cells/mL. The transfer of 100 μL of the solution (1 × 10^5^ cells) to a 5 mL culture tube was made, and 5 μL of FITC annexin V (BD, Pharmingen) and 5 μL propidium iodide (PI) were added to each tube. The cells were gently vortexed and incubated for 30 min at RT (25 °C) in the dark. Two hundred μL PBS were added to each tube. Analysis was performed with the system software (CellQuest; BD Biosciences, Mountain View, CA, USA). Lower left quadrant, viable cells (annexin V−/PI−); lower right quadrant, early apoptotic cells (annexin V+/PI−); upper right quadrant, late apoptotic cells (annexin V+/PI+); upper left quadrant, necrotic cells (annexin V−/PI+). The percentage of cells positive for PI and/or annexin V-FITC was reported inside the quadrants.

##### 3.2.2.2. Cell Cycle Analysis

The cell lines were treated with indicated concentrations of compounds. After being incubated for 48 h, cells were washed twice with ice-cold PBS, fixed and permeabilized with ice-cold 70% ethanol at −20 °C overnight. The cells were treated with 100 μg/mL RNase A at 37 °C for 30 min after washed with ice-cold PBS, and finally stained with 1 mg/mL propidium iodide (PI) in the dark at 4 °C for 30 min. Analysis was performed with the system software (CellQuest; BD Biosciences).

#### 3.2.3. Spectroscopic Studies on DNA Interaction

The 2 × 10^−3^ M ct-DNA stock solution was stored at 4 °C for no more than 5 days before use. The synthesized conjugate (**5b**) was prepared as 2 × 10^−3^ M DMSO stock solutions for DNA binding studies. The final working solutions of the complexes for DNA binding studies were diluted by TBS and the DMSO content was less than 10%. For UV-vis absorption experiments, the working solutions of the complexes were kept constant at 25 μM. The ct-DNA stock solution was increasingly added until a saturation state was achieved. After each addition, the solution was allowed to incubate for 5 min before the absorption spectra were recorded. K_b_ as the equilibrium DNA binding constant and s as the binding site size were determined by non-linear fitting according to Equation (1) [50,51]:

(1) $$\left({ɛ}_{a}-{ɛ}_{f}\right)/\left({ɛ}_{b}-{ɛ}_{f}\right)=(b-{\left(b^{2}-2K_{b}^{2}C_{t}[DNA]/s\right)}^{1/2})/2K_{b}C_{t}$$

where [DNA] is the DNA concentration in nucleotides, *ɛ*_a_ is the molar extinction coefficient of the compound bound with DNA, *ɛ*_f_ is the extinction coefficient of the free compound, and *ɛ*_b_ is the extinction coefficient of the compound fully bound to DNA. *ɛ*_a_, *ɛ*_f_ and *ɛ*_b_ are all calculated from the Lambert-Beer’s law (*ɛ = A*/[compound]). To determine *ɛ*_b_, it is assumed that the compounds are fully bound with DNA at the point where no further hypochromicity is observed under the addition of ct-DNA, and the apparent absorbance *A*_b_ is regarded as the absorbance of only the bound compounds. *C*_t_ is the constant concentration of the compounds, and *s* is the number of binding sites in base pairs. A solution containing 2 × 10^−4^ M DNA and 2 × 10^−5^ M GelRed ([DNA]/[GelRed] = 10:1) was prepared for GelRed-DNA competitive binding studies. Fluorescence emission spectra were recorded under slit width as 10 nm/10 nm for *E*_x_/*E*_m_, respectively. The quenching constant for comparing the efficiency of fluorescence quenching, *i.e.*, K_q_, of each compound was obtained by the linear fit of plotting *I*_0_/*I versus* [*Q*], according to the classic Stern-Volmer equation: *I*_0_/*I* = 1 + K_q_ × [*Q*] [58], where *I*_0_ and *I* are the peak emission intensity of the GelRed-DNA system in the absence and presence of each compound as quencher, and [*Q*] is the concentration of quencher. In the fluorescence polarization experiment, each sample was pre-incubated for 40 min before the fluorescence polarization was recorded under the condition of 595 nm emitting wavelength with 350 nm exciting wavelength, and slit width was set as 5 nm/5 nm for *E*_x_/*E*_m_, respectively. CD absorption spectra of DNA were measured in TBS at a 100 nm/min scan rate in the wavelength range from 200 to 400 nm, with 1 × 10^−4^ M DNA in the absence and presence of each compound of 2 × 10^−5^ M, respectively. The CD signal of TBS was taken as the background and subtracted from the spectra. All the spectroscopic experiments were performed at 25 °C.

### 3.3. Statistics

The data were processed by the Student’s *t*-test with the significance level *p* ≤ 0.05 using SPSS.

## 4. Conclusions

Cancer is a leading cause of death worldwide and hence studies to find anti-cancer agents for its treatment continue to grow in importance. In the present study, as potential anti-cancer agents, rhein-phosphorus derivatives were synthesized and characterized by mass spectrometry, ^1^H and ^31^P-NMR spectroscopy. It was detected that the compounds **5a** and **5b** had selective and significant cytotoxic effects on HepG-2 cells, respectively. Especially compounds **5b** exhibited the strongest cytotoxicity against HepG-2 and Spca-2 cells with IC_50_ 8.82 and 9.01 μM, respectively. In addition, it was found that **5a** and **5b** disturbed the cell cycle and induced apoptosis in HepG-2 cells and the toxic of all conjugates were lower than rhein. The binding properties of **5b** to DNA examined by various methods indicated that **5b** interacted with DNA. Therefore, these compounds may be considered as the agents with high potential anti-cancer activity and appear to be good candidates for more advanced screening.

## Supplementary Information



## Figures and Tables

**Table 1:** IC_50_^a^ values (μM) of rhein and complexes **5a**–**c** towards five selected tumor cell lines and normal cell lines for 72 h.

Compd.	R	HepG-2	CNE	Spca-2	Hela	Hct-116	HUVEC
**5a**	*p*-Ph-CH_3_	15.11 ± 1.54	33.75 ± 2.17	16.94 ± 1.32	>50	30.64 ± 8.75	>100
**5b**	Ethylbenzene	8.82 ± 0.95	27.27 ± 3.78	9.01 ± 0.87	45.36	12.66 ± 1.50	>100
**5c**	Ph	18.23 ± 1.87	38.34 ± 8.23	25.12 ± 2.72	>50	23.44 ± 3.22	>100
Rhein		38.34 ± 6.34	>50	28.31 ± 1.40	>50	>50	79.74 ± 5.40
5-Fu		20.30 ± 2.43	>50	No Date	>50	4.3 ± 0.52 [44]	No Date

a IC_50_ values are presented as the mean ± SD (standard error of the mean) from three independent experiments.
