# Supplementary material for: Synthesis, Cytotoxicity, DNA Binding and Apoptosis of Rhein-Phosphonate Derivatives as Antitumor Agents
Source: Int J Mol Sci. 2013 Apr 29;14(5):9424–39. doi: 10.3390/ijms14059424 (PMC3676791; doi:10.3390/ijms14059424)
Supplement: Supplementary file 1 [file ijms-14-09424-s001.pdf]

Supplementary Information

NMR spectra of new compounds, **5a**–**c**

A. Compound **5a**

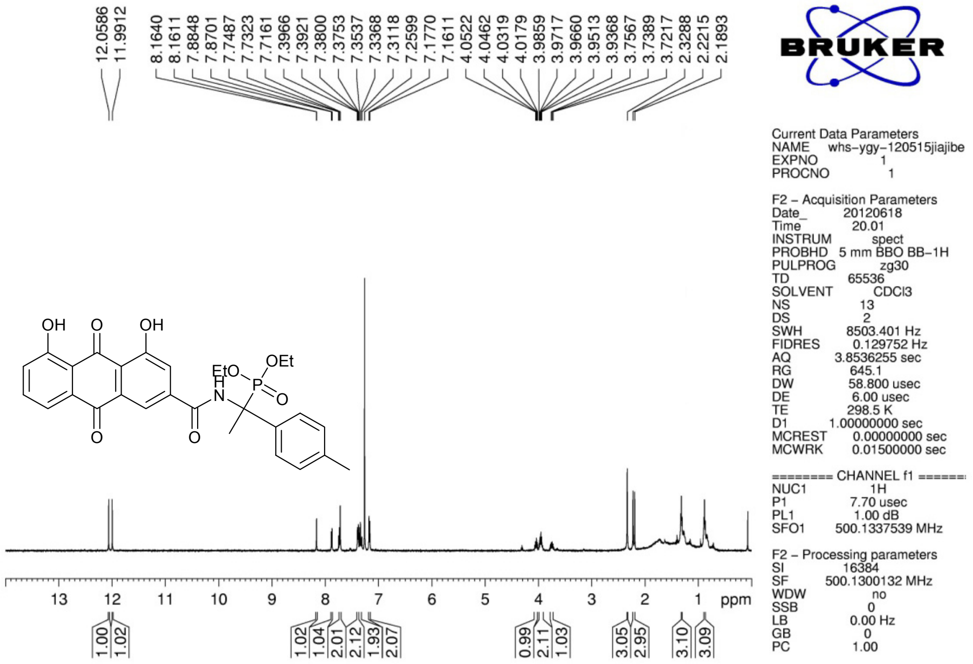

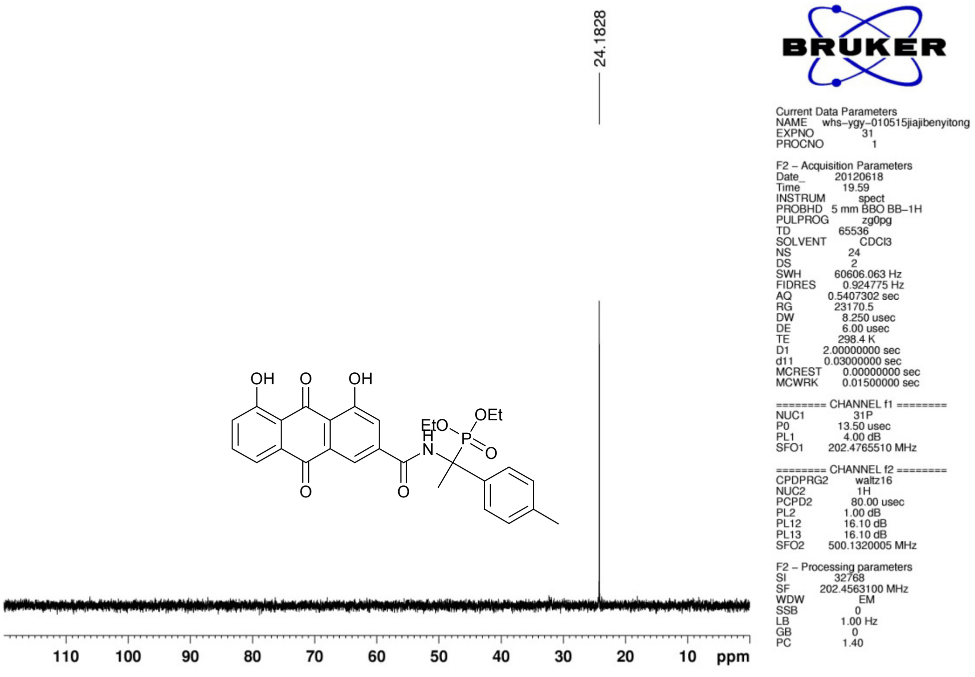

Mass Spectrum List Report

Analysis Info

Analysis Name	D:\Data\wanghengshan\yaoguiyang\ESI-whs-ygy-120515.d		
Method	aHCT_ESI_Prfile.MS.m	Operator	hct
Comment		Instrument	HCT

Acquisition Parameter

Ion Source Type	ESI	Ion Polarity	Positive	Alternating Ion Polarity	off
Mass Range Mode	Std/Enhanced	Scan Begin	300 m/z	Scan End	800 m/z
Capillary Exit	126.0 Volt	Skimmer	40.0 Volt	Trap Drive	120.3
Accumulation Time	11471 µs	Averages	10 Spectra	Auto MS/MS	off

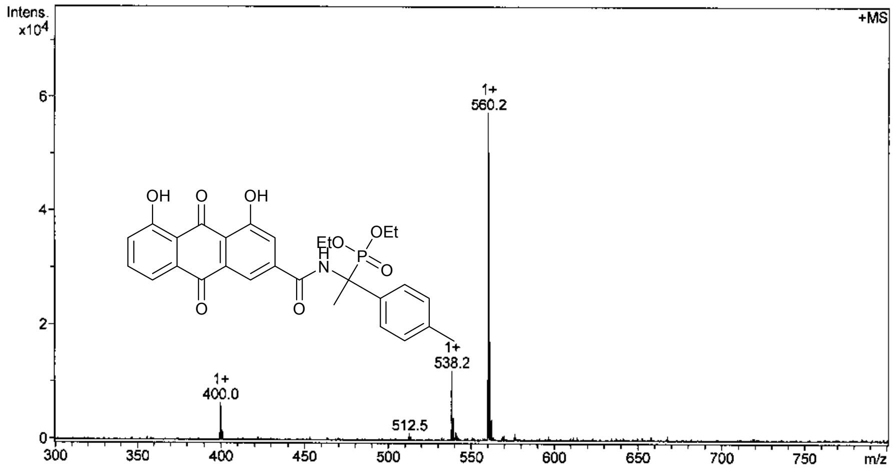

#	m/z	I
1	400.0	6319
2	401.1	1637
3	512.5	1101
4	538.2	12048
5	539.2	3868
6	540.6	1334
7	560.2	57236
8	561.2	17212
9	562.2	3504
10	576.1	3980

Bruker Daltonics DataAnalysis 3.4 Page 1 of 1

B. Compound **5b**

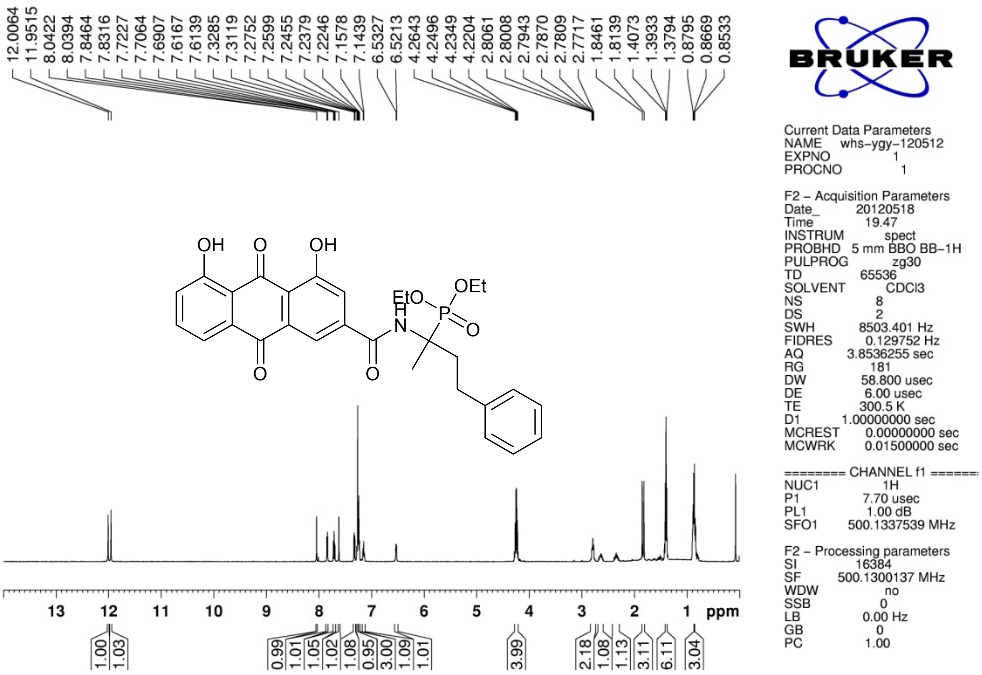

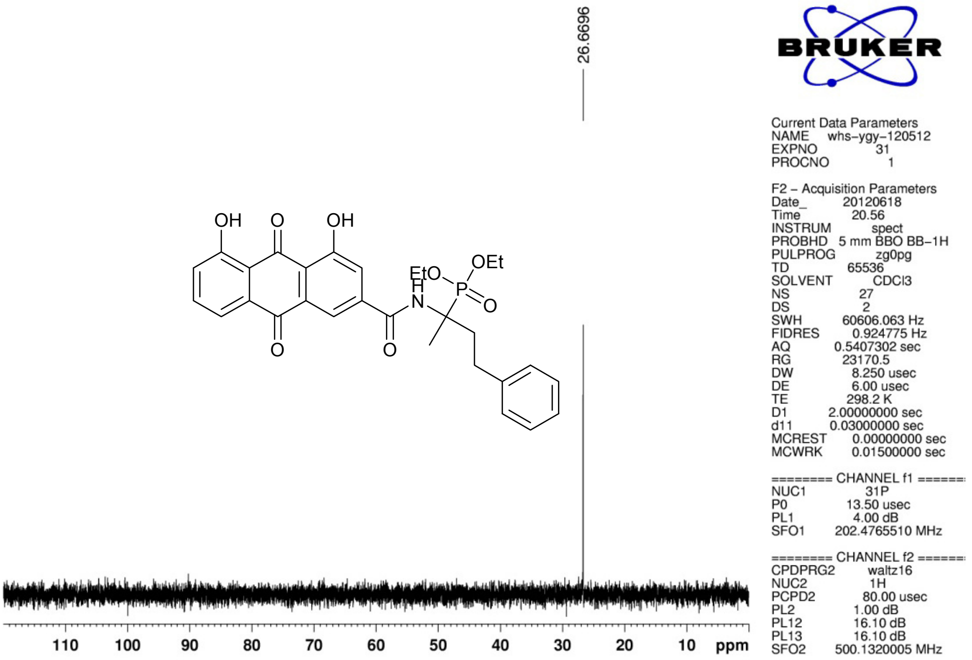

Mass Spectrum List Report

Analysis Info

Analysis Name	D:\Data\wanghengshan\yaoguiyang\ESI-whs-ygy-120512.d		
Method	aHCT_ESI_Prfile.MS.m	Operator	hct
Comment		Instrument	HCT

Acquisition Parameter

Ion Source Type	ESI	Ion Polarity	Positive	Alternating Ion Polarity	off
Mass Range Mode	Std/Enhanced	Scan Begin	300 m/z	Scan End	800 m/z
Capillary Exit	126.0 Volt	Skimmer	40.0 Volt	Trap Drive	120.3
Accumulation Time	72696 µs	Averages	10 Spectra	Auto MS/MS	off

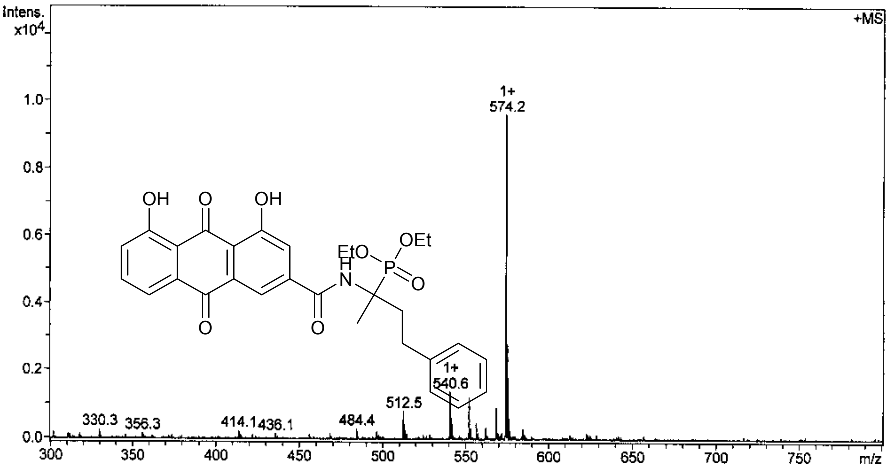

#	m/z	I
1	512.5	809
2	540.6	1382
3	541.5	620
4	552.2	1212
5	556.5	456
6	568.6	910
7	574.2	9595
8	575.2	2767
9	576.2	594
10	590.2	1064

C. Compound **5c**

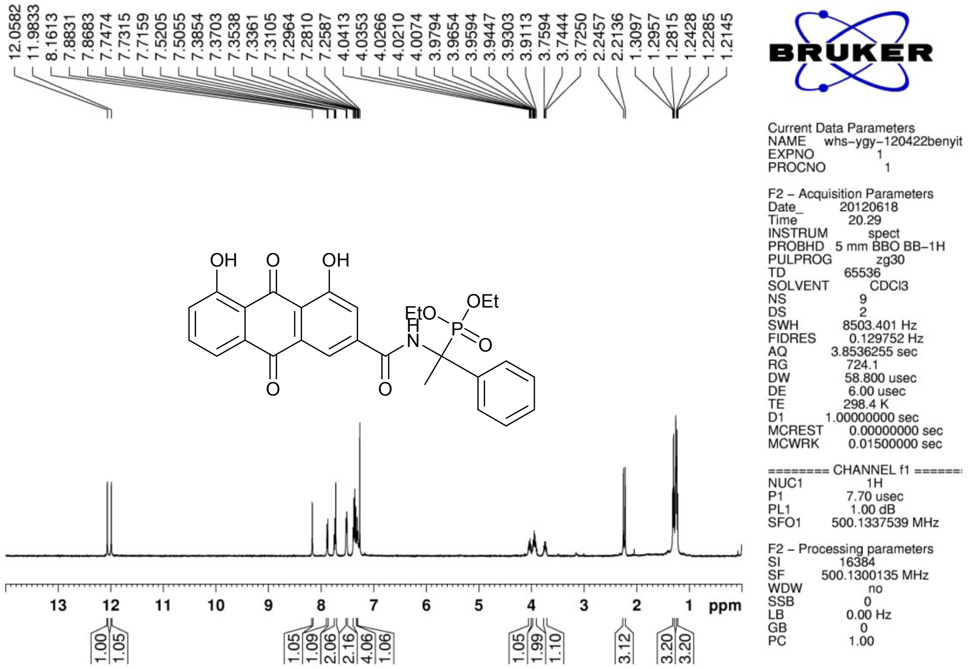

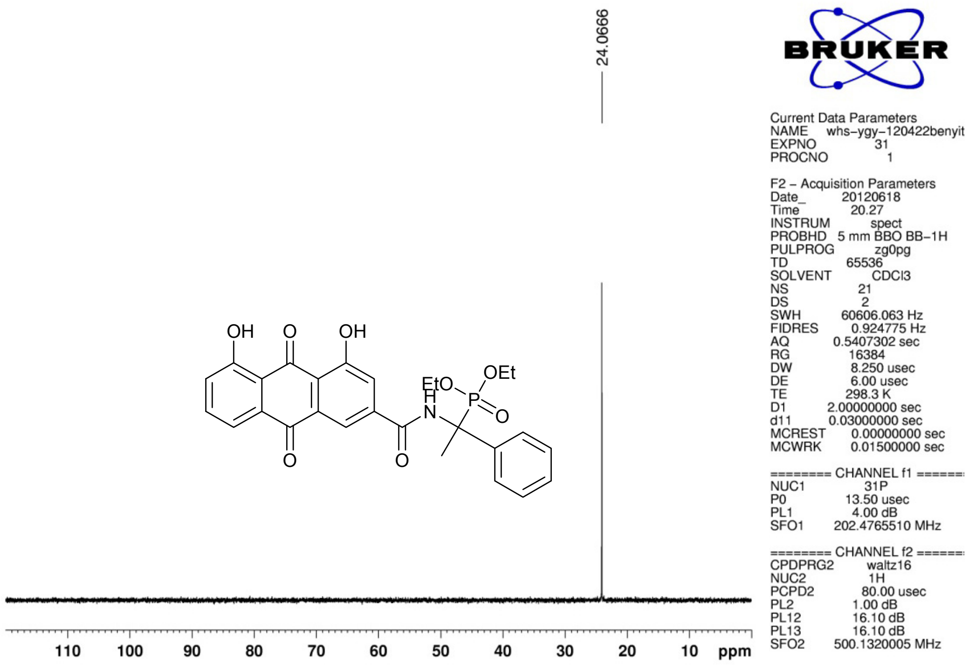

Mass Spectrum List Report

Analysis Info

Analysis Name	D:\Data\wanghengshan\yaoguiyang\ESI-whs-ygy-120422-01.d		
Method	aHCT_ESI_Prfile.MS.m	Operator	hct
Comment		Instrument	HCT

Acquisition Parameter

Ion Source Type	ESI	Ion Polarity	Positive	Alternating Ion Polarity	off
Mass Range Mode	Std/Enhanced	Scan Begin	300 m/z	Scan End	800 m/z
Capillary Exit	126.0 Volt	Skimmer	40.0 Volt	Trap Drive	120.3
Accumulation Time	2685 µs	Averages	10 Spectra	Auto MS/MS	off

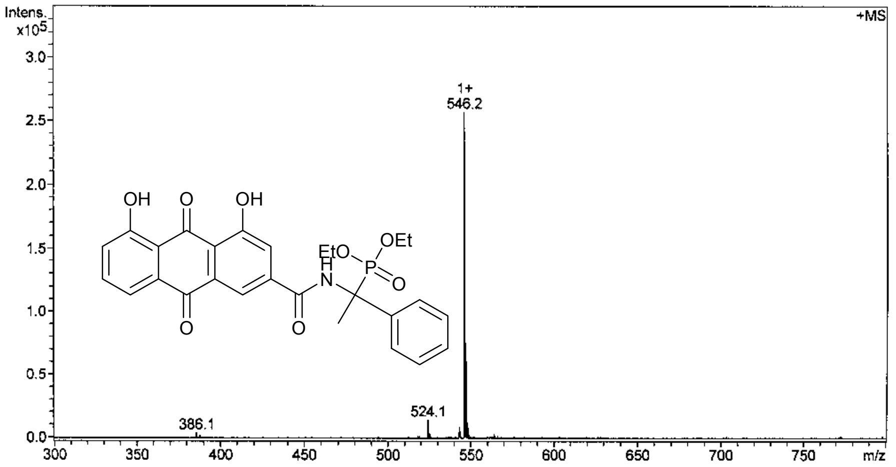

#	m/z	I
1	386.1	3727
2	524.1	14403
3	525.2	3780
4	543.1	8473
5	543.6	5184
6	546.2	256566
7	547.2	75351
8	548.2	12066
9	562.1	28998
10	563.1	8790

Figure S1. UV-Vis absorption spectra of complex **5a** in the absence (---) and presence (—) of ct-DNA with increasing [DNA]/[**5a**] ratios in the range from 1:1 to 7:1.

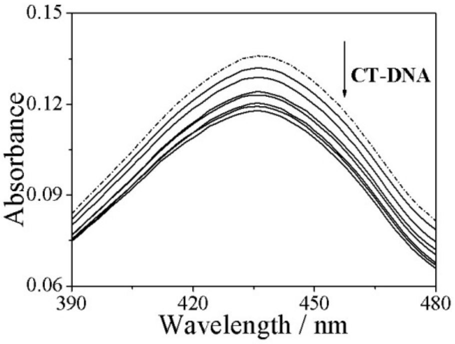

Figure S2. UV-Vis absorption spectra of complex **5c** in the absence (---) and presence (—) of ct-DNA with increasing [DNA]/[**5c**] ratios in the range from 1:1 to 7:1.

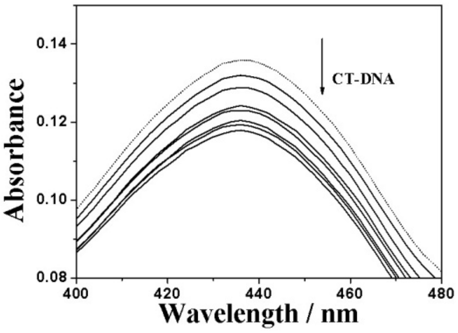

Figure S3. Fluorescence emission spectra of GelRed bound with ct-DNA ([DNA] = 2.0×10^{-3} M, [GelRed] = 2.0×10^{-3} M) in the absence (dash line) and presence (solid lines) of **5a** with [**5a**]/[GelRed] ratios range from 1:1 to 8:1. Inset: linear fitting for quenching constant K_q based on Stern-Volmer equation.

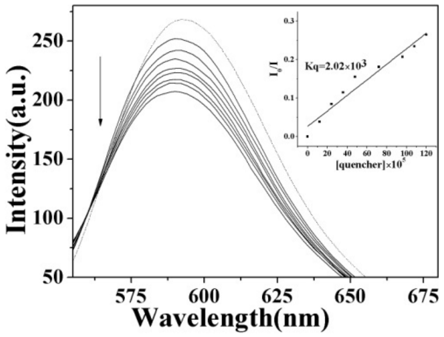

Figure S4. Fluorescence emission spectra of GelRed bound with ct-DNA ([DNA] = 2.0 × 10^{-3} M, [GelRed] = 2.0 × 10^{-3} M) in the absence (dash line) and presence (solid lines) of **5c** with [**5c**]/[GelRed] ratios range from 1:1 to 7:1. Inset: linear fitting for quenching constant K_q based on Stern-Volmer equation.

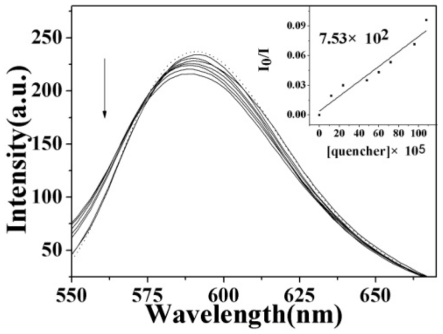

Figure S5. The quenching constant K_q of rhein.

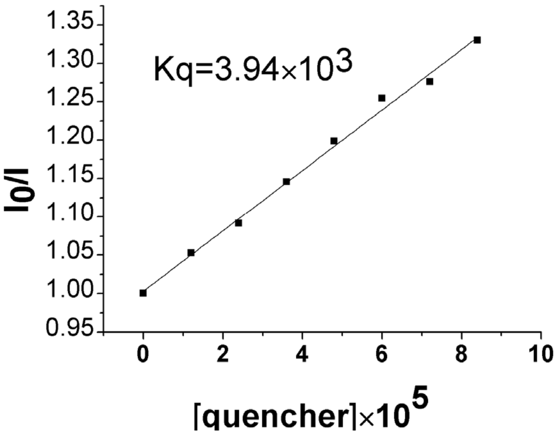

Figure S6. CD spectra of ct-DNA (2 mL solution, 1.5 × 10^{-4} M) in the absence and presence of **5a** (1.5 × 10^{-5} M).

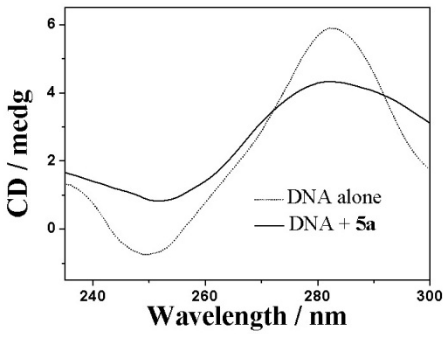

Figure S7. CD spectra of ct-DNA (2 mL solution, 1.5×10^{-4} M) in the absence and presence of **5c** (1.5×10^{-5} M).

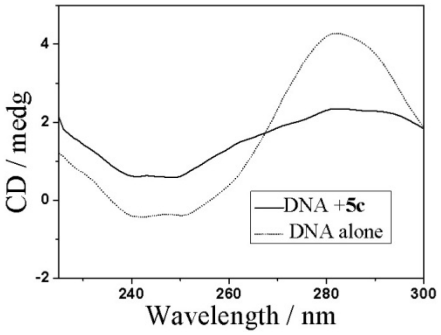

© 2013 by the authors; licensee MDPI, Basel, Switzerland. This article is an open access article distributed under the terms and conditions of the Creative Commons Attribution license (http://creativecommons.org/licenses/by/3.0/).
